# Utility of MRI Diffusion Techniques in the Evaluation of Tumors of the Head and Neck

**DOI:** 10.3390/cancers5030875

**Published:** 2013-07-05

**Authors:** José Pablo Martínez Barbero, Inmaculada Rodríquez Jiménez, Teodoro Martin Noguerol, Antonio Luna Alcalá

**Affiliations:** 1 Neuroimaging Department, SERCOSA, Health-Time Group, Carmelo Torres Street 2, 23007 Jaén, Spain; E-Mails: i.rodriguez.o@htime.org (I.R.J.); t.martin.f@htime.org (T.M.N.); 2 MRI Department, RESSALTA, Health-Time Medical Group (San Juan de Dios Hospital) Brillante Av 102, 14012 Córdoba, Spain; E-Mail: aluna70@htime.org

**Keywords:** MRI, diffusion imaging, diagnostic neuroimaging, head and neck cancer

## Abstract

The use of diffusion-weighted imaging in the head and neck is an increasingly used technique that requires adaptation of the acquisition parameters. Parallel imaging and emerging techniques such as IVIM are playing a new role. The main indications for performing DWI are tissue characterization, nodal staging and therapy monitoring. Lower apparent diffusion coefficients have been reported in this region for malignant lesions such as SCC, lymphoma and metastatic lymph node, as opposed to higher ADC in benign lesions and lymph nodes. Follow-up and early response to treatment are reflected in an ADC increase in both primary tumor and nodal metastasis.

## 1. Introduction

The head and neck are regions that present both high anatomical and functional difficulties, making the precise diagnosis and staging of regional tumors a challenging task. Many neoplasms are detected at clinical examination, but imaging techniques are also necessary for characterization of biological aggressiveness and staging. Morphological imaging techniques such as Computerized Tomography (CT) and Magnetic resonance imaging (MRI) provide anatomical information that is not always enough for the evaluation of biological characteristics of tumors in the head and neck areas. MDCT and MRI sequences provide very accurate information about tumor size and morphology, and thanks to multiplanar reconstructions (MPR) and T2 high resolution sequences, clearly depict in most cases both location and morphological characteristics of tumors in head and neck areas, The use of intravenous contrast media in both CT and MRI increases their diagnostic accuracy, and the use of T1 fat-saturated sequences is highly recommended to improve lesion detection. However, these conventional imaging techniques are limited in the evaluation of tumor response to standard chemoradiation regimens and in the detection of metastatic disease in small lymph nodes, as these techniques solely enable size measurement and shape evaluation of the lesions. Recently developed functional imaging techniques can provide information, not only on tumor size and location, but also on biological and functional aspects such as tumor vascularization and internal microarchitecture. MRI and CT, alone or combined with positron emission tomography (PET-CT), are well established methods for the initial diagnostic evaluation of neoplastic head and neck malignancies, and are also useful for the monitoring and treatment response follow up [1,2]. MR and MDCT are able to assess tumor microcirculation and viability by the use of perfusion techniques, in the same way that PET informs about the existence of hypermetabolic areas within tumors and metastasis [3,4,5]. There is controversy about the role of PET and CT in the evaluation of tumor follow-up as they use ionizing radiation, and long term follow-up studies will lead to increased total absorbed dose. Furthermore, these two imaging techniques are limited in the assessment of lesions below 1 cm. Conventional MRI provides excellent anatomical information about tumor disease in the head and neck regions, but the differentiation between residual tumor and post-treatment changes is limited. Diffusion weighted imaging (DWI) can be used to evaluate the rate of microscopic water diffusion within tissues. DWI-based techniques have been largely used to make early diagnosis and management of ischemic pathology in the brain, and later in the evaluation of white matter disorders and brain tumors. Recent technological advancements have allowed its use in the differentiation between malignant and benign lesions outside the brain. DWI may be measured by means of apparent diffusion coefficient (ADC), Areas of decreased ADC values within tumors of different regions correlate with areas of increased cellularity within tumors. Therefore, DWI has been observed as a powerful imaging biomarker of cancer [6]. Ultimately, DWI and ADC measurements are being considered as potentially useful in the evaluation and characterization of the head and neck lesions. In this article, we will further discuss the current status of DWI in the evaluation of the head and neck lesions, including technical details and clinical applications.

## 2. Physical Basis of DWI and Technical Aspects of DWI in the Neck

### 2.1. Physical Basis of DWI

**Figure 1:**
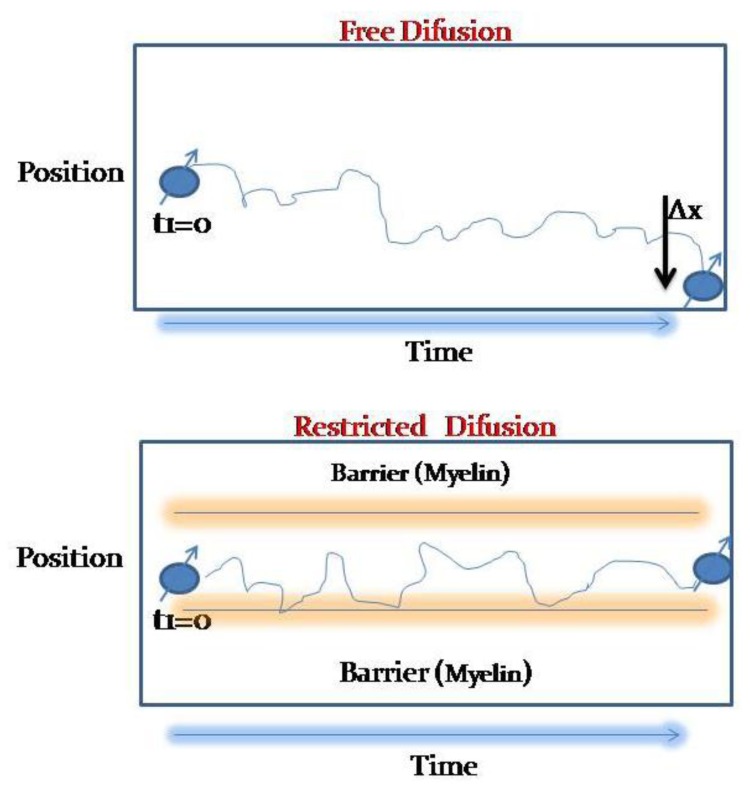
Free diffusion is described as a Brownian movement: water molecules tend to move in any space direction. In certain circumstances, like inside myelinated axons, free movement is restricted and can’t leave the intracellular space.

DWI is based on the free diffusion of water molecules due to the Brownian movement, which defines that any molecules in liquid or gaseous compounds tend to move in a random and probabilistic way in any space direction (Figure 1).

Three main mechanisms of free water movement restriction have been so far described for “*in vivo*” models [7,8]. The first described is due to restriction to the movement of water molecules between the intracellular and extracellular spaces, this mechanism is secondary to the malfunctioning of the Na/K-ATPase, which usually correlates with cellular death, and its main application is the evaluation of ischemia.

**Figure 2:**
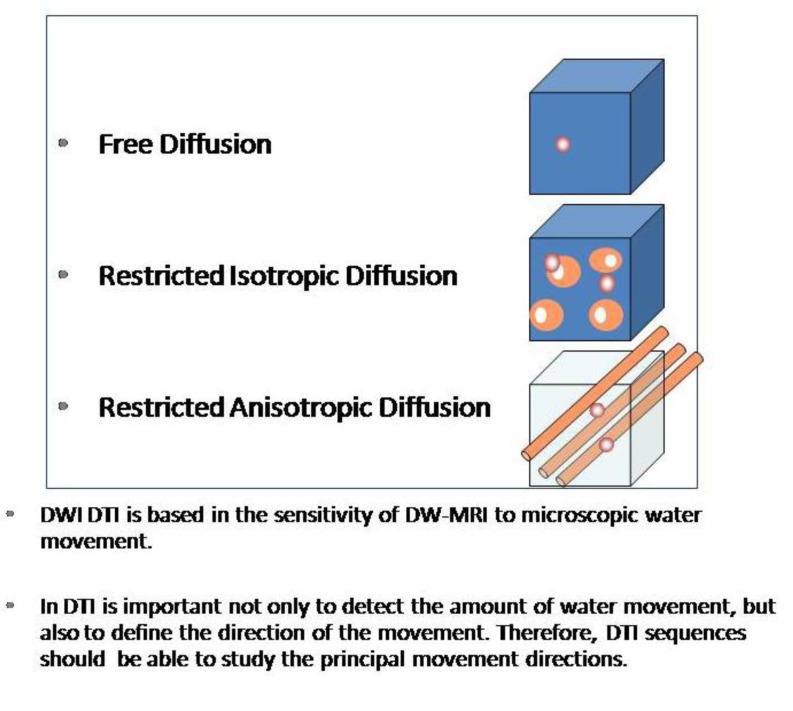
When water movement is restricted in any direction in a similar way, we will talk about isotropic restricted diffusion, but if the restriction to movement is not similar in every space direction, and the molecules tend to move easier in a certain direction, we will talk about restricted anisotropic diffusion.

The second mechanism refers to the restriction of molecular movement of inside a single cell in different directions in the space, and it has been largely used in Diffusion Tensor Imaging (DTI) studies. DTI takes advantage of this quality that water molecules have in order to approximate to the white matter structure (Figure 2).

The third major mechanism is related to the restriction of water molecules motion in situations of decreased extracellular space volume. In cancer, this mechanism may be secondary to cell growth in size or more commonly, in number. Brain tumors show a clear inverse relationship between ADC values and cellular density, so it is suggested that the high cellularity within tumors is associated with more restricted diffusivity. In that way, ADCs values are expected to vary according to the microstructures of tissues or pathologic states as it has been shown in other regions, including the head and neck areas. ADC values correlate with tumor cellularity, and can be used as an independent biomarker in HHNSCC [9].

In addition, DWI “*in vivo*” is modified by cellular packing, intracellular and extracellular components and macromolecules, so DWI provides insights of a cellular scale. Sensibility to diffusion-based contrast is controlled by appropriate b-value range (multiexponential signal decay).

Nowadays, the processes involved in diffusion changes at a cellular level are not properly known. Thus, a better understanding of DWI is needed in order to establish the real biophysical properties of water movement within the cellular matrix.

### 2.2. Technical Aspects of DWI in the Neck

The performance of DWI in the head and neck regions is technically demanding due to the presence of air-bone-soft tissue interfaces, involuntary or deglutition motions (breathing or swallowing), physiologic vascular pulsation and dental fillings with metallic materials. DWI is vulnerable both to motion and magnetic susceptibility artifacts since the majority of DWI studies based on Eco Planar Imaging (EPI) sequences that usually have also limited spatial resolution. Therefore, scanning parameters of the DWI sequence must be optimized in order to increase SNR (signal to noise ratio) and CNR (contrast to noise ratio). DWIBS (diffusion-weighted imaging with background body subtraction) may also be used in the head and neck regions, although it suffers from important anteroposterior distortion and the ADC values obtained with this technique are lower than the ones obtained with conventional EPI-DWI [10].

In order to reduce motion artifacts, the reduction of the acquisition time appears as an interesting and necessary option. This reduction should be carried out with a maximum scan time of 5 min as a good reference. The DWI-Periodically Rotated Overlapping Parallel lines with Enhanced Reconstruction (PROPELLER) acquisition reduces motion-related artifacts, as it benefits from a modified radial acquisition scheme with rotating parallel lines, which inherently oversamples the k-space center and can be used to correct in-plane motion. This approach is also insensitive to susceptibility artifacts and hemorrhage and offers better conspicuity for lesions located in the air-tissue interface, although it has limited SNR and requires longer acquisition times. These shortcomings are even more pronounced at 3T, as distortion and field inhomogeneity at tissue boundaries increase in comparison with 1.5T [11].

The quantification of DWI becomes important for lesion characterization in the head and neck areas. Recent studies have shown that with modern DWI acquisition techniques, ADC values obtained are independent of the magnetic field strength, despite the fact that some older studies showed that ADC values could change depending on the magnet field strength [9,12]. As it has been described for other organs, ADC values are limited since they are influenced by tissue perfusion. This effect is more evident with low b values. Intravoxel Incoherent Motion (IVIM) analysis of DWI signal decay allows differentiating between true diffusion from perfusion [13,14]. Calculation and quantification of IVIM-derived parameters, such as D* (perfusion contribution to signal decay), D (real diffusion of water molecules) and *f* (perfusion contribution to the diffusion signal) are being tested for the evaluation of several diseases and organs. There is growing data suggesting that D is a more reliable marker of tissue diffusion than ADC [15]. In fact, several tests have already proved that ADC equals D in the absence of tissue perfusion. However, *in vivo*, these trials have verified that ADC values are higher than expected, due to blood microcirculation, therefore, it is called *apparent* diffusion coefficient. In addition, IVIM shows the potential to assess tissue microcirculation and vascularization without the use of contrast media, using parameters as D* and *f*. Different correlation levels between these parameters and dynamic-contrast-enhanced MRI and histological markers of neoangiogenesis have been reported for primary brain malignancies, liver metastasis, pancreatic, rectal and prostatic carcinoma [15,16,17]. Therefore, IVIM derivated parameters, could be used, in a near future as potential biomarkers for the monitoring and follow up of treatment response in head and neck cervical oncological conditions. Initial data suggest that an initial high *f* may predict poor prognosis in HNSCC [18] and that different head and neck tumors may have distinctive *f* and D values [19]. In order to increase the DWI sequence quality several rules should be followed (Table 1).

**Table 1:** DWI technical aspects.

Minimize T1 saturation	TR (repetition time) should be long enough to avoid T1 saturation effects
**Use short TE (echo time)**	Increasing the bandwidth (up to a maximum of 1,500 MHz) using parallel imaging (SENSE factor up to 2) and by increasing the gradient intensity in the gradient lobes
**Increase the number of adquisitions (NEX)**	Noise is disruptive and the signal is additive
**Decrease the field of view (FOV)**	Minimum in phase encoding direction.
**Do not increase the resolution in plane to levels where the noise increases**	It will decrease the quality of ADC maps; enlarging the FOV may have a similar result
**Use fat supression sequences**	Increase the dynamic range of the DWI

### 2.3. Measurement Approach of ADC Values in the Head and Neck

There is a great variability in the proposed ADC threshold values, according to the intensity of themagnetic field (1.5 T or 3T), the number of b values selected for the ADC calculation, the maximum b value used or, of course, the type of cancer. Another important source of variability in the ADC values depends on how and where the region of interest (ROI) has to be placed inside the lymph node [20]. Due to the intrinsic design of the DWI sequence, the use of fat suppression (spectral or not) causes a decrease in signal intensity of lymph node, especially in the fat hilum area. Then, lower ADC values than expected will be obtained, giving a false positive; as the presence of a preserved fatty hilum is a morphological criterion of benignity. However, other studies have shown significantly differences when the fatty hilum is included, as it would induce an overestimation of real ADC value of lymph nodes [21]. Nevertheless is clear that hilum should be excluded when a ROI is placed for ADC measurement of lymph nodes.

In metastatic nodes with necrosis, ADC values would be higher than expected if those necrotic areas, with high diffusivity due to liquefactive or coagulative necrosis, are included, leading to a false negative measurement. The use of a histogram analysis approach for the mensuration of ADC values is recommendable to assess the node heterogeneity and its standard deviation for an adequate screening of metastatic cells clusters inside the lymph nodes.

One of the other sources of variability present in ADC cutoff values described in literature is the different size of the region of interest (ROI) when ADC measures are performed. As a general rule, ROI should have at least the same volume as the voxel of the DWI-sequence to avoid both positive or negative false results due to partial volume average effects [20].The ROI should include the entire node perimeter, avoiding both hilum and necrotic areas, using a free handed drawn setting. That approach, along with the histogram data analysis will lead to a better understanding of the whole node microstructure.

In concern to the *b* values used, a *b* value of 800–1,000 s/mm^2^ would provide a good spatial resolution and an adequate signal/noise ratio for lymph nodes evaluation. The use of *b* values over 1,000 s/mm^2^ would offer better contrast but are more prone to suffer susceptibility artifact. On the other hand, the use of *b* values lower than 300 s/mm^2^ will lead to overestimated ADC values, due to the influence of the perfusion effect of small vessels.

Another problem that should be taken into consideration while making ADC measurements is the inter-observer reliability, as in follow-up studies probably many readers will make measurements. A recent study showed that EPI-DWI detects more lesions than HASTE-DWI, and that both present good inter-observer agreement; but the agreement between those two techniques for ADC values was not good enough [22].

## 3. Clinical Applications of MRI-DWI in Head Neck Neoplastic Lesions

### 3.1. Detection and Evaluation of Primary Malignancies of the Upper Respiratory Tract and Malignant Lymph Nodes

**Figure 3:**
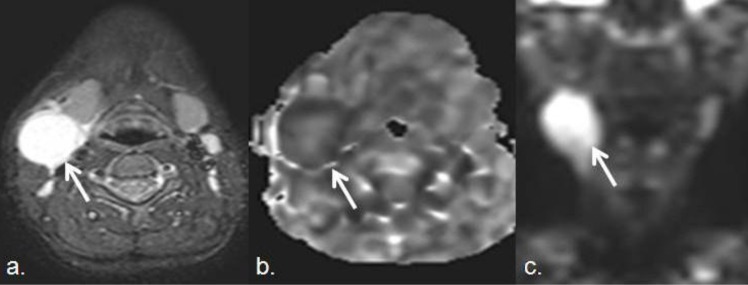
Thirty seven year old female. She had a growing lymph node at left III level, after and oral infection. (**a**) Transverse STIR shows an enlarged lymph node in the right internal jugular chain (white arrow). (**b**)The lymph node demonstrates intermediate signal on ADC map (ADC value 2.5 × 10^−3^), and high signal on DWI with high b value (**c**), due toT2-shine through effect and absence of restricted diffusion. Percutaneous biopsy proved a benign inflammatory origin secondary to an ENT infection.

**Figure 4:**
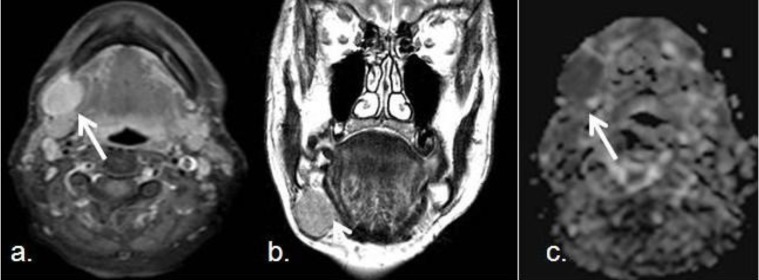
Lymphoma. (**a**) Transverse STIR and (b) coronal TSE T2-weighted images show an enlarged right submandibular lymph node. (**c**) Decreased signal of this lymph node (white arrow) on the ADC map (ADC value 0.69 × 10^−3^ mm^2^/s) confirms high restriction of free water diffusion, that supports the diagnosis of lymphoma (histologically proven).

Characterization and differentiation between neoplastic and non-neoplastic lymph nodes is one of the main purposes for performing imaging techniques in head and neck cancer studies [19]. Metastatic adenopathies can be detected in imaging studies better than in clinical examination even in inaccessible locations such as retropharyngeal or paratracheal lymph chains. Imaging also plays an important role for the monitoring of tumor response and for detecting recurrent or persistent disease before it becomes clinically evident [23]. SCC (squamous cell carcinoma) is the most common type of tumor in the oral cavity, and it usually spreads through the lymphatic system to cervical nodes. Studies have revealed that the higher the grading, the higher the risk of cervical metastasis [24]. Lymph nodes metastasis in the neck with occult primary is a recognized clinical entity. In 81.1% of the cases, SCCs are responsible for this disease, and adenocarcinomas account for 7.6% of cases, the majority of those cases belong to primary tumors outside the neck [23]. Morphological criteria in both CT and MRI for characterization of lymph nodes are limited in the neck region. Overlap of the imaging characteristics of metastasis, lymphoma and benign inflammatory or infectious lymph node are common. Functional information derived from DWI can increase lesion detection and help to make this distinction, especially if malignancy is an issue. The differences between ADC values of head neck lymph nodes according to their different origin have been well documented in literature [25]. ADC values are usually lower in nodal lymphomas than in metastatic lymph nodes from SCC with a suggested cut-off point around 0.76 × 10^−3^ mm^2^/s, according to several series [26,27]. ADC of highly and/or moderately differentiated SCC is greater than the one of poorly differentiated ones, which may show overlap in their ADC values with lymphoma [26]. Benign lymph nodes usually demonstrate ADC higher than lymphomas, commonly in a range over 1.4 × 10^−3^ m/s^2^ to 2.5 × 10^−3^m/s^2^ [28] (Figure 3, Figure 4).

Furthermore, DWI allows an accurate post-treatment monitorization of both primary tumors and nodal metastasis. Recently, it has also been proposed as a proper way to predict response to therapy [29]. Primary tumors with lower ADC values are more likely to have a complete response to chemoradiation. This is due to the absence of hypoxia, one of the main mechanisms of radio-resistance. In a similar way, metastatic lymph nodes with low ADC values also generally show a better response to the treatment. According to initial results using the IVIM-approach, it seems likely that lower baseline ADC-values in patients with HNSCC and good short term outcome are partly caused by perfusion effects (*f*-value).

**Figure 5:**
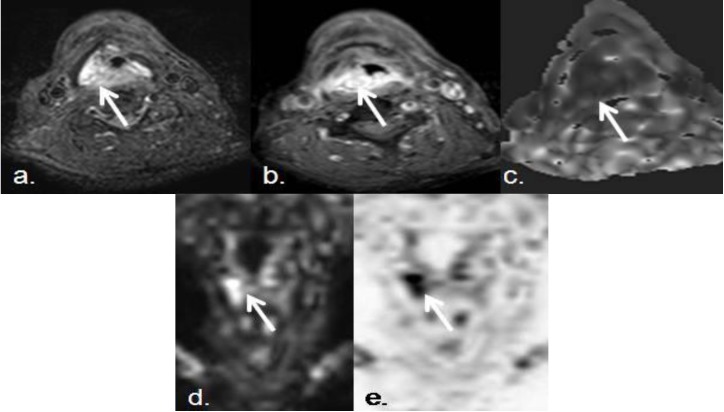
Seventy two year old male, with squamous cell carcinoma of the right piriform sinus.Despite his small size, this malignant lesion associated marked diffusion restriction. (**a**,**b**) Pre- and postcontrast axial SPIR TSE T1-weighted images show a small enhancing and infiltrating lesion in right piriform sinus (white arrows); (**c**) Axial ADC map, (**d**) DWIBS coronal MIP, and (**e**) DWIBS inverted MIP (pseudo-PET) show high increased signal in DIBWS sequence and decreased signal in ADC map, that confirms diffusion restriction of the lesion (ADC: 0.8 × 10^−3^ mm^2^/s). DWI and ADC can be considered in routine clinical practice as complementary sequences to anatomical ones, in order to increase diagnostic accuracy.

Sometimes, in the characterization of primary tumors in the head and neck tumors, conventional MRI and CT are not specific enough, The standardized use of DWI sequences in this area is showing promising results to improve their diagnostic accuracy. It has been well documented the usefulness of the ADC values in the characterization and differentiation of both SCC and lymphoma [30,31]. It has been well established that mean ADC values of SCC were significantly larger than those in lymphomas [29]. From a histological point of view, lymphomas may have characteristically more cellularity, larger nuclei with more macromolecular proteins and less extracellular space than well or moderately differentiated squamous cell carcinomas. The distinction from the very poorly differentiated carcinomas is however more difficult to establish. DWI provides information about tumor cellularity, and advanced techniques such as IVIM give information related to tumor microperfussion [13]. Moreover, some authors propose ADC values of 0.76 × 10^−3^ mm^2^/s, with an accuracy of 98%, to recognize the difference between these tumors [32]. The ADC values in metastatic nodes from SCC may be variable according with histological tumor variations, but are lower than benign lesions (Figure 5).

**Figure 6:**
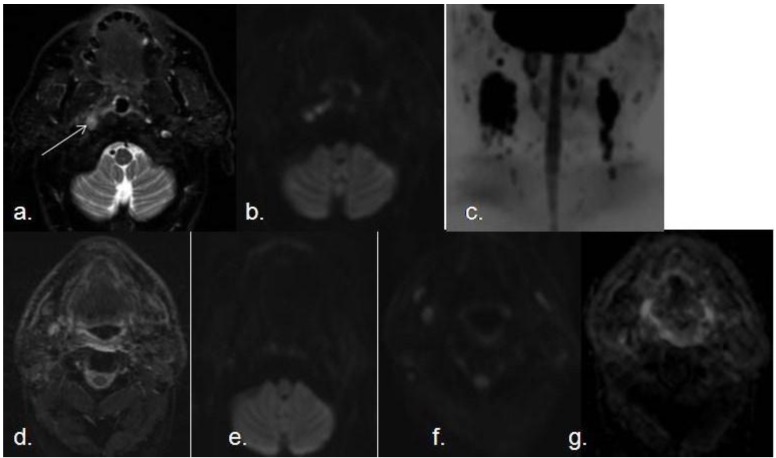
Pre- and post-reatment SCC with lymph node metastasis. (**a**) Transverse STIR shows a small nodular hyperintense lesion in right torus tubarius; (**b**) The lesion demonstrates increased signal intensity on DWI with high b value (white arrow); (**c**) Coronal MIP of a DWIBS sequence with high b value demonstrates several bilateral enlarged lymph nodes with restricted diffusion The lesion was biopsied and confirmed as a SCC with bilateral metastatic lymph nodes. Postreatment follow-up MRI (six months after radio and chemotherapy); (**d**) axial STIR; (**e** and **f**) axial DWI with high b value at two different levels; (**g**) corresponding ADC map, demonstrates absence of recurrence, with no areas of restricted diffusion or suspicious lymph nodes.

Furthermore, ADC values may not only be useful to distinguish between benign and malignant pathologies in their pretherapeutic state but they also can be useful to identify recurrent tumors after therapy. This is possible because DWI can be used in addition to anatomic studies in routine monitorization of tumor response and tumor follow up. Vandecaveye *et al*. and others [33,34] found significant differences between ADC values of persistent or recurrent head and neck squamous cell carcinoma from non tumoral post-radiation changes (Figure 6).

### 3.2. Salivary Gland Tumors, Orbitary and Pediatric Lesions

**Figure 7:**
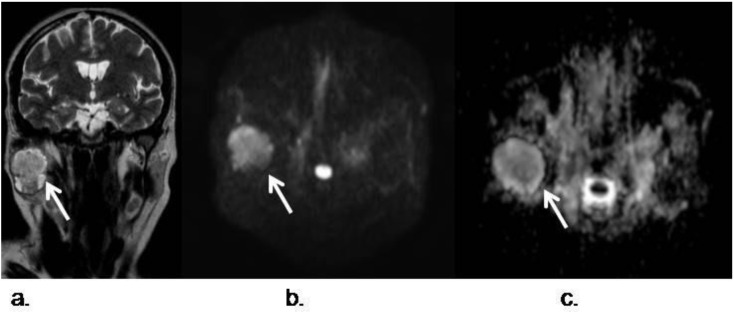
Pleomorphic adenoma of the right parotid gland; (**a**) Coronal TSE T2-weighted image shows a right intraparotid well defined lesion (white arrow); (**b**) Transverse DWI acquired with a *b* value of 800 s/mm^2^ demosntrates hyperintensity of the lesion which is also hypointense in the corresponding ADC map (**c**), indicating restriction of free water diffusion. The mean ADC value was 1.6 × 10^−3^ mm^2^/s. In this case, DWI favors pleomorphic and helps to distinguish it from a Warthin tumor.

Salivary gland tumors are uncommon, but this complicated anatomical location requires usually an accurate pre surgical diagnosis to correctly select the less aggressive and more efficient treatment [23]. In general, benign lesions show higher ADC values than malignant ones, but some overlap has been described as, some benign processes associate low ADC values and aggressive mimicry [35]. This is particularly possible in reactive lymph node without macro necrosis and Whartin tumors [36,37]. In the case of reactive lymph nodes, a large accumulation of inflammatory cells with lymphoid germinal centers and varying amount of stromal fibrosis cause restriction of the free diffusion of the extracellular water protons and lead to low ADC values. However, a great proliferation rate of the epithelial component and lymphoid accumulation in the stroma may cause restriction in the motion of the water protons in the extracellular space of the Warthin tumor and the high cellularity of glandular cells may be the cause for the low ADCs of parotid hyperplasia. Because of that, particular care must be taken when assessing the ADCs from tumors of the salivary and parotid glands [38]. Pleomorphic adenomas usually show high ADC values (between 1.5 and 2 × 10^−3^ mm^2^/s). Conversely, Warthin tumors demonstrate low ADC values (between 0.7 and 1.1 × 10^−3^ mm^2^/s). This is probably because of their different histologic origin, as pleomorphic adenomas show a heterogeneous component of epithelial, myoepithelial and stromal cells with areas of fluid within the epithelial glandular areas, and Warthin tumors have a lymphoid origin with decreased extracellular space. In addition, acinic cell carcinomas of the parotid glands show intermediate ADC values between pleomorphic adenomas and Warthin tumors, as they are hypercellular lesions, but not as much hypercellular than Warthin tumors (Figure 7).

Another potential application of DWI is in the one related to the differential diagnosis of cystic lesions according to ADC measurements, including congenital cystic lesions and abscesses or infectious-necrotic lesions at any age. The free water diffusion is compromised by intracellular structures, presence of macromolecules or fibers, thus it is no surprise that the restriction in the diffusivity is lower in benign cystic lesions than of solid lesions. Fewer differences in ADC values among cystic lesions are caused by protein component variations in the fluid and the changes in the viscosity of the contents.DWI techniques are especially indicated for characterizing lesions in children because the highest incidence of congenital entities like development cysts and other processes in head and neck, and for differential diagnosis with malignant tumors, that are oftenly very aggressive at these ages. Cystic lesions of head and neck can be routinely diagnosed with conventional MRI sequences, but in certain situations such as infected branchial cleft cysts or epidermal cysts, DWI can play an important complementary help. Epidermal cysts typically associate restriction in diffusion and low ADC values and are located frequently in the head and neck at first decade [39]. However, there is still controversy over whether DWI can play a role in the differentiation between vascular anomalies like hemangiomas and aggressive soft-tissue tumors such as rhabdomyiosarcoma [40].

**Figure 8:**
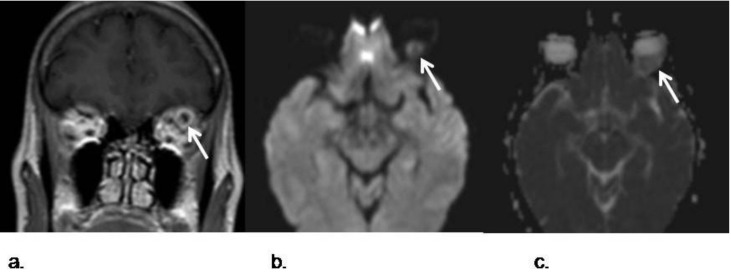
Intraorbitary metastasis of retroperitoneal leiomyosarcoma. (**a**) Postcontrast coronal TSE T1-weighted image of the orbits shows a left intraorbital nodule in the intraconal space. The lesions shows a ring-like pattern of enhancement with a central hypointense area. This appearance was not specific, as many masses in the orbit may have similar presentation, like an abscess or tumors; (**b**) DWI with a *b* value of 1,000 s/mm^2^; (**c**) corresponding ADC map show a marked restriction of the lesion that favours malignancy. Posterior surgical removal confirmed a metastasis from a retroperitoneal leiomyosarcoma.

There is growing interest in the use of DWI for evaluation of orbital masses and optic nerve pathologies, such as ischemic or inflammatory neuritis. The performance of DWI in the orbit is especially challenging, but DWI appears as interesting when improving detection of malignant lesions, such as as metastasis or orbitary lymphoma, which usually show low ADC values [41,42] (Figure 8).

## 4. Conclusions

DWI is now technically feasible in the head and neck regions. The dddition of DWI to MRI protocols in the evaluation of head and neck malignancies increases lesion detection and helps the differentiation process between both solid and cystic lesions and benign from malignant lesions. Reported data suggests a role for ADC measurements in the noninvasive characterization of lymph node in the head and neck region. Furthermore, recent series have claimed a role for DWI in primary tumor follow-up, in the evaluation of treatment response and in the detection of local recurrency. The application of more complex model analysis, such as IVIM, may increase DWI accuracy and provide information of lesion perfusion without the use of contrast agents.
